# Factors influencing harmonized health data collection, sharing and linkage in Denmark and Switzerland: A systematic review

**DOI:** 10.1371/journal.pone.0226015

**Published:** 2019-12-12

**Authors:** Lester Darryl Geneviève, Andrea Martani, Maria Christina Mallet, Tenzin Wangmo, Bernice Simone Elger

**Affiliations:** 1 Institute for Biomedical Ethics, University of Basel, Basel, Switzerland; 2 Institute of Social and Preventive Medicine, University of Bern, Bern, Switzerland; 3 University Center of Legal Medicine, University of Geneva, Geneva, Switzerland; Karolinska Institutet, SWEDEN

## Abstract

**Introduction:**

The digitalization of medicine has led to a considerable growth of heterogeneous health datasets, which could improve healthcare research if integrated into the clinical life cycle. This process requires, amongst other things, the harmonization of these datasets, which is a prerequisite to improve their quality, re-usability and interoperability. However, there is a wide range of factors that either hinder or favor the harmonized collection, sharing and linkage of health data.

**Objective:**

This systematic review aims to identify barriers and facilitators to health data harmonization—including data sharing and linkage—by a comparative analysis of studies from Denmark and Switzerland.

**Methods:**

Publications from PubMed, Web of Science, EMBASE and CINAHL involving cross-institutional or cross-border collection, sharing or linkage of health data from Denmark or Switzerland were searched to identify the reported barriers and facilitators to data harmonization.

**Results:**

Of the 345 projects included, 240 were single-country and 105 were multinational studies. Regarding national projects, a Swiss study reported on average more barriers and facilitators than a Danish study. Barriers and facilitators of a technical nature were most frequently reported.

**Conclusion:**

This systematic review gathered evidence from Denmark and Switzerland on barriers and facilitators concerning data harmonization, sharing and linkage. Barriers and facilitators were strictly interrelated with the national context where projects were carried out. Structural changes, such as legislation implemented at the national level, were mirrored in the projects. This underlines the impact of national strategies in the field of health data. Our findings also suggest that more openness and clarity in the reporting of both barriers and facilitators to data harmonization constitute a key element to promote the successful management of new projects using health data and the implementation of proper policies in this field. Our study findings are thus meaningful beyond these two countries.

## Introduction

Technological advances made over the past few years have increased the digitalization of medicine, thus leading to a considerable growth of clinical, research and public health datasets. These data sources are increasingly related to the *big data* environment and they include, amongst others, genomics and other-omics related-data collections, electronic health records (EHRs), patient registries, medical imaging, administrative data and clinical trials data [1, 2]. However, a good part of such datasets are often kept and analysed in silos and not adequately shared [3]. If properly integrated into the clinical life cycle, such collections of data stand to offer a unique opportunity to drive scientific discoveries and improve healthcare research. For example, they may allow a better understanding of the aetiology of illnesses and subsequently help in improving the management, prevention and treatment of diseases [1, 2]. This is even more promising in the framework of learning healthcare systems, where clear boundaries between research and care are dissolving and the same data are used both for improving scientific knowledge and providing better care [4].

In this context, developing the harmonization of health data—described as the sum of all “efforts to combine data from different sources and provide users with a comparable view of data from different studies” [5]—is crucial to improve clinical research and practice. Such standardized approach requires not only better quality, re-usability and interoperability of data, but also more open and collaborative communication between the different stakeholders active in the health data environment [6]. The fact that a good percentage of healthcare spending are being wasted as a consequence of under-exploiting data potential in several healthcare systems around the world [7–9] should be considered as one important factor urging for such changes to happen. Harmonized health datasets are also laying the foundation of a new era of biomedical research, where three concepts are currently converging, namely precision medicine, learning healthcare systems and implementation science [7, 10].

The harmonization of health data is a complex procedure which involves significant changes in how data are collected, shared and linked. Harmonization can be either prospective, when modifications occur in the study design to subsequently render the pooling of data more straightforward, or retrospective, when pooling is performed with data collected previously according to different study designs [11]. In practical terms, harmonization can be achieved through two distinct but complementary approaches, namely a “stringent” and a “flexible one” [12]. By means of a “stringent” approach, data are harmonized through the use of standard collection tools and standard operating procedures, implementable only in a prospective way. With the “flexible” approach, on the contrary, different data collection tools might be used, as long as operating procedures are standardized [12].

In achieving the harmonization of health data, careful consideration needs to be given to already well-known as well as novel challenges related to the processes of collection, sharing and linkage. Such challenges are drastically intensified by the vastness and the hyper-connectedness of data at present time [13], which may result in unforeseen connections or cross-referencing between datasets, drastically increasing re-identification risks for data subjects [14]. The presence of these challenges has resulted in the emerging of several barriers to the effective use and sharing of health-related data [2, 15]. Although these have been categorized as technical, motivational, economic, political, socio-cultural, ethical and legal [1, 2, 15–17], a more precise mapping of the exact content of such barriers, and of the solutions that have been elaborated to mitigate them, is lacking.

Within this framework, the aim of this systematic review is to identify more precisely some of the barriers and facilitators encountered in the effort to achieve harmonization of health data—including the processes of data sharing and linkage—by a comparative analysis of studies conducted in two countries having different healthcare systems and data infrastructures, namely Denmark and Switzerland. These countries where chosen because, although they both offer high quality healthcare, they have two very diverse healthcare systems and two different data infrastructure models for healthcare. Denmark has a Beveridge-based national healthcare system [18] and a long tradition of data linkage in health through its nationwide registries [19]. On the contrary, Switzerland is based on a federalist Bismarckian organization of healthcare [20, 21] and started much later to develop strategies in the field of Health Information Exchange [22]. In this perspective, this review seeks to identify past and current studies related to the field of harmonized health data collection, sharing and linkage in these two countries and list the barriers encountered and the facilitators that make these projects successful. Furthermore, the review aims to provide some insights on the complexities associated with the use of health data that can be of relevance also in the broader international context.

## Methodology

### Search strategy and study selection

This study conformed to the Preferred Reporting Items for Systematic Reviews and Meta-Analysis (PRISMA) guidelines [23], and its protocol was registered on January 3^rd^ 2018 on PROSPERO (CRD42018081424). A systematic literature search was performed on four search engines and electronic bibliographic databases namely PubMed, Web of Science (all databases), EMBASE (no Medline) and CINAHL for publications with dates ranging from 1^st^ January 2008 to 31^st^ December 2017. The time period is aligned with the adoption of the Swiss national eHealth Strategy in 2007, with the aim of introducing electronic patient records at national level [24]. The search was repeated for the period of 1^st^ January 2018 to 31^st^ March 2019 to include additional publications and to ensure that our systematic review is up-to-date. Reference lists of included publications were screened to identify other potential harmonized health data collection, sharing or linkage projects. A search strategy was developed for each electronic database. The literature search included Medical Subject Headings (MeSH) terms and free applicable text to health data collection, sharing and linkage. The search strategy consisted of three components, namely (1) types of health data, (2) keywords for data collection, sharing and linkage and (3) country of interest. For instance, the search strategy for Switzerland on PubMed was: ("Administrative Claims, Healthcare"[Mesh] OR "Health Records, Personal"[Mesh] OR "Clinical Coding"[Mesh] OR "Patient Discharge Summaries"[Mesh] OR "Clinical Trials as Topic"[Mesh]) AND ("Databases as Topic"[Mesh] OR "Data Collection"[Mesh] OR "Medical Informatics"[Mesh] OR "Medical Record Linkage"[Mesh] OR "Information Dissemination"[Mesh] OR "Data Integration" OR "Data Sharing") AND ("Switzerland"[Mesh]) [filters used are Articles types (Clinical Study, Clinical Trials (including controlled and Phases I to IV), Comparative Study, Evaluation Studies, Journal Article, Multicenter Study, Observational Study, Pragmatic Clinical Trial, Randomized Control Trial, Technical Report and Validation Studies), language (Danish, English, French and German) and species (Human Studies)]. We did not include harmonization as an imperative component in our search strategy since the exact boundaries of this concept are still controversial [25] and the addition of the term “harmonization” as an imperative component drastically reduced the number of publications for each country.

Eligibility criteria for this study were: (i) publications based on health data collection, sharing or linkage projects. There was no restriction on study design and type, i.e. qualitative, quantitative or mixed method studies, and clinical or observational studies were included; systematic reviews were excluded; (ii) there were no restriction on age, gender, disease and ethnic group of participants involved in these studies; (iii) the studies had to involve some health data collection, sharing or linkage at cross-institutional, cross-national or cross-regional levels in at least one of the two countries; (iv) only English, French, German and Danish language articles were included, and (v) publication year of articles ranged from January 2008 to March 2019.

### Data extraction and quality assessment

The literature search results were catalogued on EndNote^TM^ X8, a reference manager software. The titles and abstracts of all articles were screened independently by two authors (LDG and AM). The full-texts of the included publications were reviewed by LDG and AM to ensure that they met the eligibility criteria to be included in the systematic review. LDG and AM performed independently the data extraction from the included articles through a standard data extraction form developed progressively by the authors of this review. Additional publications gathered through reference screening went through title and abstract, and independent full-text screenings and data extraction by MCM. Another review author, TW, validated randomly twenty percent of the publications reviewed by LDG, AM and MCM, to assess the quality of the data extraction process. A disagreement level of less than 10% for the data entries was considered acceptable.

The data extraction form included (i) study information (author(s) and publication year), (ii) sources of health data, (iii) cross-institutional or cross-national nature of the study, (iv) presence or absence of primary and secondary health data collection, analysis and sharing, and lastly (v) the categorization of barriers and facilitators to harmonized health data collection, sharing and linkage. The sources of health data were categorized as having three standard origins, namely health services, public health and research [26]. Other sources of health data falling outside these three categories were classified in a residual category (“Other”).

LDG and AM performed a categorization of the identified barriers and facilitators separately, and came to consensus on the final categorization of these elements for accuracy and inclusiveness. Disagreements were solved with the mediation of TW. The identified barriers and facilitators to harmonized health data collection, sharing and linkage were subsequently clustered into main categories, which were then sub-clustered into smaller categories to highlight the most common barriers and facilitators in these main categories (the full clustering/sub-clustering of barriers and facilitators is shown in Table 1). For the purpose of this systematic review, we defined harmonization techniques as methods which would allow the coherent pooling of different data sources, involving health data collected either prospectively, retrospectively or both. Examples include the use of standard case report forms or data dictionaries, a central review of the collected data, training provided to researchers/stakeholders and leadership role by one of the partners for coordinating data collection, sharing or linkage activities.

**Table 1:** Clustering of barriers and facilitators to harmonized health data collection, sharing and linkage.

Barriers		Facilitators	
Cluster	Sub-cluster	Cluster	Sub-cluster
**Ethical**	Privacy	**Ethico-Legal**	Ethical approval by REC/IRB
	Respect for Autonomy		Health Data Anonymization
	Other		Informed Consent
**Legal**	Data Protection Regulations		Patient data access rights
	Divergence in National Legislations for Data Security and Privacy		Confidentiality measures
	Other		Clarity of legislation for health data collection/sharing/linkage
			Official/legal approval of project
			Study according to International laws and regulations
			Legislation allows project without consent or REC approval
			Legislation requires mandatory reporting
			Other
**Technical**	Lack of Data Standards (data structure and semantics)	**Technical**	Data harmonization techniques
	Data Quality Issues		Data Linkage techniques
	Limited Technical Capabilities		
	Other		Other
**Financial**	Lack of Funding	**Financial**	Securing funding
	Other		Public-Private partnership
			Other
**Political**	Mistrust between stakeholders	**Political**	Data Sharing Agreement
	Data Ownership		Building and maintaining stakeholder trust
	Institutional/constitutional organization issues		Data access control
	Other		Health System Structure
			Other
**Motivational**	Lack of research incentives	**Motivational**	Monetary Incentive
	Stakeholder restricts access for re-use of data as deemed unfit for secondary use		Easing workload through improvement of data collection
	Stakeholder competing interests		Memorandum of understanding to ensure collaboration until end of study
	Other		Other
**Sociocultural**	Cultural clash for data collection/sharing/linkage	**Sociocultural**	Participant data access control
	Other		Other

### Analysis

A narrative synthesis of included publications was carried out [27]. This step involved the categorization of health data collection, sharing and linkage projects based on their national or cross-national dimension, their source of health data, and barriers and facilitators identified in these publications. This step was important to highlight similarities and differences between projects in Denmark and Switzerland. The statistical software, STATA ® version 15.0, was used for the different analyses.

## Results

**Fig 1:**
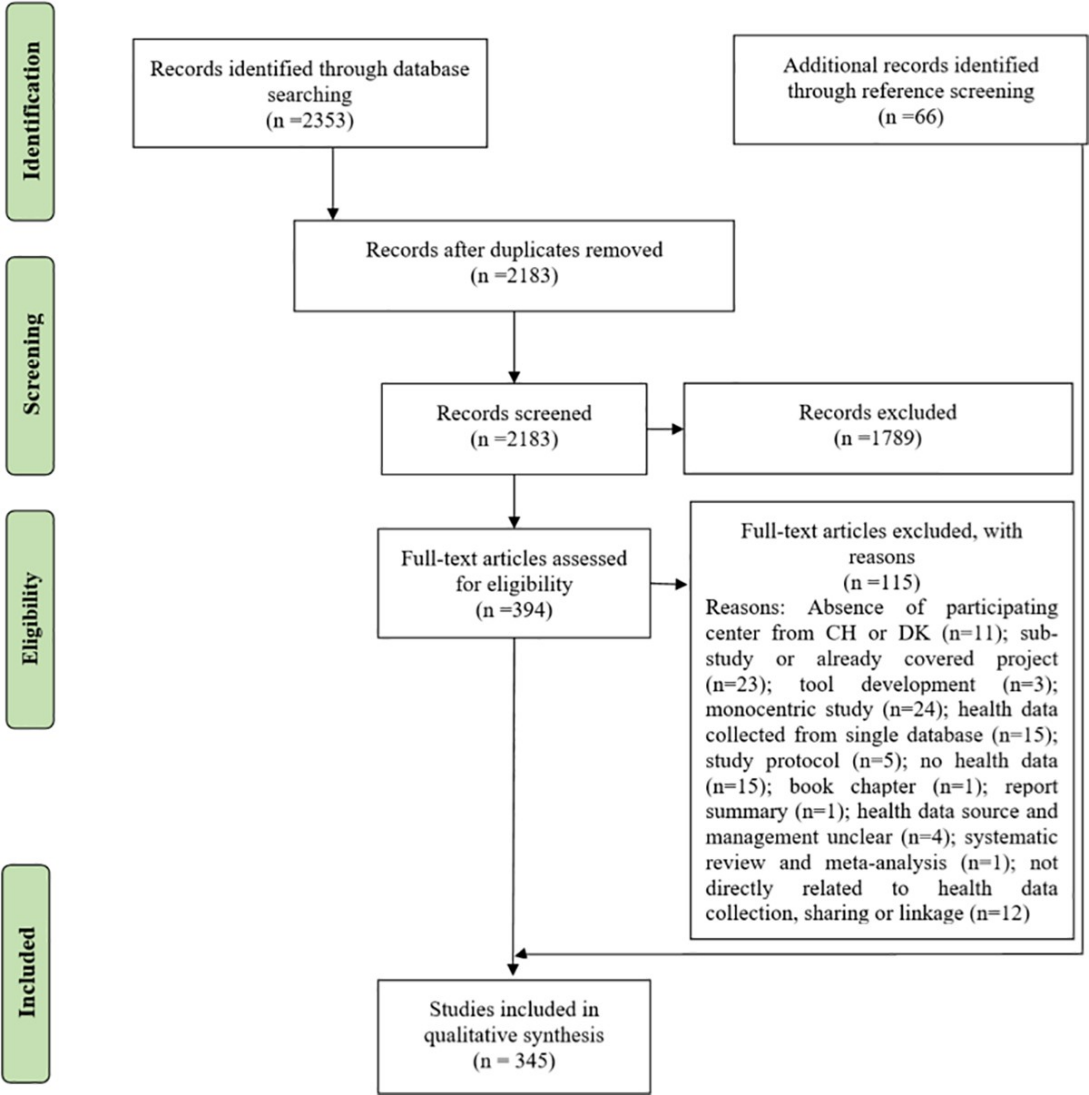
Flow diagram of study selection.

A total of 1928 papers were initially retrieved from the search engines and electronic bibliographic databases for the period of January 2008 to December 2017. The search was repeated for the period of January 2018 to March 2019 (upon request of the journal) resulting in a total of 425 additional papers. The result of the two searches were combined for each stage of the PRISMA resulting in an overall total of 2353 papers retrieved for the period of January 2008 to March 2019 (Fig 1). Duplicates (n = 170) were removed either automatically using ENDNOTE X8 or manually after reviewing abstracts and their titles. The remaining 2183 papers went through title and abstract screening, which resulted in the exclusion of 1789 papers. In-depth full-text screening was performed for 394 papers, and 115 more papers were excluded for not meeting the inclusion criteria (see Fig 1 for reasons). Reference screening of the 279 included papers, resulted in the identification and inclusion of 66 additional papers which met the eligibility criteria for this systematic review (Fig 1).

The 345 included papers are summarized in Table 2, where they are categorized based on their national (n = 240) or cross-national (n = 105) dimension, their sources of health data and the total number of barriers and facilitators reported for each project. We identified 200 Danish and 40 Swiss national projects, and 105 cross-national projects. Among these cross-national projects, 14 projects involved the use of health data from both Denmark and Switzerland, 51 and 40 projects involved a Danish partner and a Swiss partner respectively. Overall, the number of projects which involved primary health data collection, sharing and analysis were 106 (30.7%), 92 (26.7%) and 106 (30.7%) respectively. Comparatively, the number of projects which involved secondary health data collection and analysis were 283 (82.0%; if a study collected both primary health data and secondary health data, it was counted for both). Of the 345 projects, 199 used health data from health services, 211 from public health sector, 94 from research and 62 from other health data sources.

**Table 2:** Characteristics of included projects (n = 345) with the total number of identified barriers and facilitators per project.

Reference	Country	Partnership Type	Source of health data	Total Identified Barriers, n	Total Identified Facilitators, n
Aabakke et al. 2014 [28]	DK^a^	National	Health Services; Public Health; Other	4	5
Adam et al. 2010 [29]	CH^b^	National	Research	5	7
Agergaard et al. 2017 [30]	DK	National	Health Services	3	6
Agten et al. 2017 [31]	CH	National	Public Health	1	3
Ammundsen et al. 2012 [32]	DK	National	Health Services; Research	3	6
Andersen et al. 2011 [33]	DK	National	Health Services; Public Health	4	8
Andersen et al. 2014 [34]	DK	National	Health Services, Public Health	2	4
Andres et al. 2018 [35]	CH	National	Public Health	4	3
Annaheim et al. 2018 [36]	CH	National	Health Services; Other	5	3
Antonsen et al. 2011 [37]	DK	National	Public Health; Research	2	2
Antonsen et al. 2016 [38]	DK	National	Health Services; Public Health; Research	2	12
Arboe et al. 2016 [39]	DK	National	Health Services; Public Health; Other	0	14
Arking et al. 2014 [40]	CH	Cross-national	Research	0	4
Atladóttir et al. 2012 [41]	DK	National	Health Services; Public Health; Other	1	4
Aubert et al. 2016 [42]	CH	National	Health Services	0	5
Auer et al. 2014[43]	CH	National	Health Services; Public Health	0	9
Avillach et al. 2013 [44]	DK	Cross-national	Health Services; Public Health	5	12
Avlund et al. 2018 [45]	DK	National	Health Services; Public Health	1	8
Bachelet et al. 2016 [46]	DK	Cross-national	Health Services; Public Health; Research	2	7
Baker et al. 2009 [47]	DK	National	Health Services	3	2
Baldur-Felskov et al. 2013 [48]	DK	National	Health Services; Research	2	5
Balgobind et al. 2009 [49]	DK	Cross-national	Health Services; Research	0	1
Bay-Nielsen et al. 2008 [50]	DK	National	Public Health	0	2
Beduneau et al. 2017 [51]	CH	Cross-national	Health Services; Public Health	1	6
Begre et al. 2010 [52]	CH	National	Other	2	4
Bendixen et al. 2019 [53]	DK	National	Health Services; Public Health; Research; Other	0	3
Beretta-Piccoli et al. 2017 [54]	CH	National	Health Services; Public Health	3	8
Binderup et al. 2018 [55]	DK	National	Health Services; Research; Other	2	5
Bisgaard et al. 2013 [56]	DK	National	Health Services; Research	0	5
Bjerregaard and Larsen 2011 [57]	DK	National	Health Services; Public Health	0	11
Bjornholt et al. 2015 [58]	DK	National	Health Services; Public Health; Research	1	9
Blaha et al. 2016 [59]	DK	Cross-national	Health Services	2	6
Blenstrup and Knudsen 2011 [60]	DK	National	Health Services; Research	1	3
Blichert-Toft et al. 2008 [61]	DK	National	Health Services; Public Health; Research	0	3
Bodin et al. 2018 [62]	DK	Cross-national	Health Services; Public Health	3	6
Boje et al. 2014 [63]	DK	National	Public Health	1	3
Brenner et al. 2011 [64]	CH	National	Public Health	1	6
Brink et al. 2018 [65]	DK	National	Research	5	6
Burgstaller et al. 2016 [66]	CH	National	Health Services; Research	0	6
Cainzos-Achirica et al. 2018 [67]	DK	Cross-national	Health Services	4	5
Calhaz-Jorge et al. 2017 [68]	DK	Cross-national	Public Health; Other	2	4
Calvet et al. 2014 [69]	CH	Cross-national	Research	2	3
Carstensen et al. 2008 [70]	DK	National	Health Services	0	4
Caspersen et al. 2008 [71]	DK	National	Health Services; Public Health	0	7
Chaigne et al. 2017[72]	CH	National	Health Services	0	5
Chesnaye et al. 2014 [73]	DK	Cross-national	Public Health	1	2
Chmiel et al. 2011 [74]	CH	National	Health Services	3	10
Christensen et al. 2011 [75]	DK	National	Health Services; Public Health; Research	0	2
Christensen et al. 2011b [76]	DK	National	Public Health	0	11
Christensen et al. 2011c [77]	DK	National	Health Services; Public Health	1	5
Christensen et al. 2014 [78]	DK	National	Health Services; Public Health; Other	1	8
Christensen et al. 2016 [79]	DK	National	Public Health	1	6
Christensen et al. 2016b [80]	DK	National	Health Services; Public Health	1	6
Christiansen et al. 2008 [81]	DK	National	Public Health; Research	1	5
Christiansen et al. 2008b [82]	DK	National	Health Services; Public Health	1	3
Christoffersen et al. 2015 [83]	DK	National	Health Services; Public Health; Other	1	5
Coleman et al. 2011 [84]	DK	Cross-national	Public Health	2	13
Coloma et al. 2011 [85]	DK	Cross-national	Health Services	5	9
Corraini et al. 2017 [86]	DK	National	Public Health	1	6
Costantino et al. 2018 [87]	DK	Cross-national	Health Services; Other	4	6
Cotter et al. 2013 [88]	CH	Cross-national	Health Services; Research	1	3
Czauderna et al. 2016 [89]	CH	Cross-national	Research	4	9
Dalgard et al. 2010 [90]	DK	Cross-national	Research	0	0
Damgaard et al. 2013 [91]	DK	National	Public Health	1	3
Darby et al. 2013 [92]	DK	Cross-national	Health Services; Public Health	1	4
Dastani et al. 2012 [93]	CH	Cross-national	Research	0	4
De Angelis et al. 2009 [94]	Both	Cross-national	Research	5	7
De Groot et al. 2014 [95]	DK	Cross-national	Health Services; Public Health; Research	4	1
della Torre et al. 2012 [96]	CH	National	Health Services	0	5
Dencker et al. 2016 [97]	DK	National	Public Health	1	1
De Vos Andersen et al. 2017 [98]	DK	National	Health Services; Public Health; Other	2	9
Diel et al. 2010 [99]	CH	National	Health Services	3	7
Disanto et al. 2016 [100]	CH	National	Public Health	0	11
Donia et al. 2017 [101]	DK	National	Public Health; Research	2	0
Downs et al. 2016 [102]	DK	Cross-national	Public Health; Other	1	2
Dreyer et al. 2015 [103]	DK	Cross-national	Public Health; Other	2	7
Edgren et al. 2015 [104]	DK	Cross-national	Health Services; Public Health; Other	1	6
Ehlers et al. 2009 [105]	DK	National	Public Health; Other	0	3
Ekelund et al. 2015 [106]	DK	National	Health Services; Public Health	1	12
El-Galaly et al. 2015 [107]	DK	Cross-national	Public Health	1	5
Elliott et al. 2017 [108]	DK	National	Health Services	1	2
Engelberger et al. 2015 [109]	CH	National	Health Services; Public Health	0	6
Erdem et al. 2015 [110]	DK	Cross-national	Health Services; Other	1	2
Erichsen et al. 2010 [111]	DK	National	Health Services; Public Health; Research	0	13
Erichsen et al. 2011 [112]	DK	National	Health Services; Public Health	1	4
Erlangsen et al. 2008 [113]	DK	National	Public Health	1	5
Escala-Garcia et al. 2019 [114]	DK	Cross-national	Research	2	2
Escott-Price et al. 2014 [115]	CH	Cross-national	Research	0	0
Fagö-Olsen et al. 2012 [116]	DK	National	Public Health	0	1
Fahrner et al. 2014 [117]	CH	National	Health Services	4	2
Fedder et al. 2013 [118]	DK	National	Public Health	2	5
Fenger et al. 2016 [119]	DK	National	Public Health; Other	1	5
Fieten et al. 2018 [120]	CH	Cross-national	Research	3	3
Fløe et al. 2018 [121]	DK	National	Health Services; Other	1	4
Frandsen et al. 2014 [122]	DK	National	Health Services; Public Health	2	5
Frary et al. 2016 [123]	DK	National	Health Services; Public Health	0	5
Freiberg et al. 2017 [124]	Both	Cross-national	Health Services; Public Health	0	3
Friis et al. 2009 [125]	DK	National	Health Services; Public Health; Other	1	4
Funcke et al. 2016 [126]	CH	Cross-national	Health Services; Public Health	1	7
Furtwängler et al. 2018 [127]	CH	Cross-national	Health Services; Research	1	4
Gammelager et al. 2012 [128]	DK	National	Health Services; Public Health	1	7
Garcia-Etienne et al. 2019 [129]	CH	Cross-national	Health Services	1	4
Gatta et al. 2017 [130]	CH	Cross-national	Research	1	1
Gatzioufas et al. 2016 [131]	CH	Cross-national	Research	0	4
Geissbuhler 2013 [132]	CH	National	Health Services	16	15
Ghith et al. 2012 [133]	DK	National	Research; Other	2	7
Gjerstorff 2011 [134]	DK	National	Public Health	1	7
Glintborg et al. 2011 [135]	DK	National	Health Services; Public Health	2	5
Godballe et al. 2009 [136]	DK	National	Public Health	0	5
Gorski et al. 2015 [137]	CH	Cross-national	Research	1	6
Goutaki et al. 2017 [138]	Both	Cross-national	Health Services	2	14
Goutaki et al. 2019 [139]	CH	National	Health Services; Research	4	10
Gradel et al. 2008 [140]	DK	National	Health Services; Public Health	0	5
Grann et al. 2011 [141]	DK	National	Health Services; Public Health	1	6
Gratwohl et al. 2015 [142]	CH	Cross-national	Research	0	4
Gregersen et al. 2016 [143]	DK	National	Public Health	3	6
Griffin et al. 2011 [144]	DK	Cross-national	Health Service; Research; Other	2	3
Gromov et al. 2014 [145]	DK	National	Health Services	0	5
Gruber et al. 2018 [146]	CH	Cross-national	Health Services	0	2
Gudbrandsdottir et al. 2012 [147]	DK	National	Health Services; Other	1	1
Gulmez et al. 2009 [148]	DK	National	Health Services; Public Health	0	4
Gylvin et al. 2017 [149]	DK	National	Health Services; Research; Other	1	3
Hallas et al. 2012 [150]	DK	National	Health Services; Public Health	3	6
Hallas and Pottegard 2017 [151]	DK	National	Health Services; Public Health	1	5
Halmin et al. 2017 [152]	DK	Cross-national	Public Health	0	4
Hansen et al. 2008 [153]	DK	National	Health Services; Public Health	1	8
Hansen et al. 2012 [154]	DK	National	Health Services; Public Health	2	4
Hansen and Jacobsen 2014 [155]	DK	National	Health Services; Research	1	6
Hansen et al. 2018 [156]	DK	National	Health Services; Research; Other	2	4
Harshman et al. 2012 [157]	DK	Cross-national	Health Services; Public Health	1	2
Hatz et al. 2011 [158]	CH	National	Public Health	1	6
Haueis et al. 2012 [159]	CH	Cross-national	Research	1	4
Havelin et al. 2009 [160]	DK	Cross-national	Public Health	3	6
Head et al. 2013 [161]	DK	Cross-national	Health Services; Other	3	5
Helgstrand et al. 2010 [162]	DK	National	Health Services; Public Health	0	7
Helgstrand et al. 2012 [163]	DK	National	Health Services; Public Health; Other	0	3
Helqvist et al. 2012 [164]	DK	National	Health Services; Public Health	0	3
Helweg-Larsen 2011 [165]	DK	National	Public Health; Other	1	3
Hemkens et al. 2017 [166]	CH	National	Research	0	5
Henningsen et al. 2011 [167]	DK	National	Public Health	0	4
Henningsen et al. 2011b [168]	DK	Cross-national	Public Health	4	8
Henriksen et al. 2013 [169]	DK	National	Public Health	0	3
Herzberg et al. 2012 [170]	DK	National	Health Services	1	3
Hetland 2011 [171]	DK	National	Health Services; Other	5	16
Holland-Bill et al. 2014 [172]	DK	National	Health Services; Public Health	1	8
Horsdal et al. 2012 [173]	DK	National	Health Services; Public Health	2	5
Hyldig et al. 2019 [174]	DK	National	Health Services; Public Health; Research; Other	1	5
Ingeholm et al. 2016 [175]	DK	National	Health Services; Public Health; Other	2	6
Ittermann et al. 2018 [176]	DK	Cross-national	Research	1	4
Iversen et al. 2016 [177]	DK	National	Public Health	1	8
Jacobs et al. 2014 [178]	CH	Cross-national	Research	0	11
Jakobsen et al. 2017 [179]	DK	National	Public Health	2	2
Jensen et al. 2009 [180]	DK	National	Health Services; Public Health	1	6
Jensen et al. 2010 [181]	DK	National	Health Services; Public Health	1	0
Jensen et al. 2011 [182]	DK	National	Health Services	2	6
Jensen et al. 2016 [183]	DK	National	Public Health	1	7
Jensen et al. 2017 [184]	DK	National	Public Health	1	1
Jeppesen et al. 2016 [185]	DK	National	Health Services; Public Health	2	5
Johannesdottir et al. 2012 [186]	DK	National	Health Services; Public Health	3	9
Jørgensen et al. 2018 [187]	DK	National	Health Services; Public Health	1	6
Joshi et al. 2015 [188]	Both	Cross-national	Research	1	6
Kachuri et al. 2018 [189]	DK	Cross-national	Research	0	2
Kaltoft et al. 2009 [190]	DK	National	Health Services; Public Health	2	3
Karkov et al. 2010 [191]	DK	National	Health Services; Public Health; Other	2	5
Kent et al. 2015 [192]	DK	National	Health Services; Public Health; Other	1	13
Khanna et al. 2008 [193]	CH	National	Research	1	1
Khatami et al. 2016 [194]	Both	Cross-national	Health Services	2	14
Kiderlen et al. 2012 [195]	CH	Cross-national	Public Health	3	2
Kildemoes et al. 2011 [196]	DK	National	Health Services; Public Health	1	8
Kirwan et al. 2008 [197]	Both	Cross-national	Research	2	12
Klein et al. 2012 [198]	DK	National	Health Services; Public Health	1	1
Knudsen et al. 2013 [199]	DK	National	Health Services	0	1
Kowalska et al. 2011 [200]	DK	Cross-national	Health Services; Other	4	4
Kronborg et al. 2009 [201]	DK	National	Public Health; Other	1	6
Laenkholm et al. 2018 [202]	DK	National	Health Services; Public Health	0	8
Laguna et al. 2009 [203]	CH	Cross-national	Health Services	0	3
Landolt et al. 2016 [204]	CH	Cross-national	Research	0	3
Lang et al. 2019 [205]	CH	Cross-national	Research	2	3
Lange et al. 2017 [206]	DK	National	Health Services; Other	0	5
Laouali et al. 2018 [207]	DK	Cross-national	Public Health; Other	1	6
Larsen et al. 2016 [208]	DK	National	Public Health; Research	2	3
Larsen et al. 2016b [209]	DK	National	Health Services; Public Health	3	5
Laursen et al. 2018 [210]	DK	National	Health Services; Public Health; Research	1	5
Leboeuf-Yde et al. 2012 [211]	DK	National	Research; Other	1	1
Lehnert et al. 2018 [212]	DK	National	Public Health	1	3
Lildballe et al. 2014 [213]	DK	National	Health Services; Public Health	0	2
Linauskas et al. 2018 [214]	DK	National	Public Health	7	4
Lindhardsen et al. 2011 [215]	DK	National	Health Services	1	7
Lindhardsen et al. 2012 [216]	DK	National	Health Services; Other	2	8
Linnet et al. 2009 [217]	DK	National	Health Services; Public Health; Other	2	7
Liu et al. 2016 [218]	DK	National	Health Services; Public Health; Research	1	7
Lund et al. 2018 [219]	DK	National	Public Health	4	8
Lundstrøm et al. 2009 [220]	DK	National	Public Health	3	5
Luta et al. 2018 [221]	CH	National	Research	0	6
Lydiksen et al. 2014 [222]	DK	National	Health Services; Public Health	0	3
Lynge et al. 2011 [223]	DK	National	Health Services	3	4
Maeng et al. 2008 [224]	DK	National	Health Services; Public Health	1	4
Mahajan et al. 2018 [225]	DK	Cross-national	Research	2	6
Majholm et al. 2012 [226]	DK	National	Health Services; Public Health	3	3
Mareri et al. 2011 [227]	Both	Cross-national	Research	0	4
Margulis et al. 2017 [228]	DK	Cross-national	Public Health	2	5
May et al. 2014 [229]	CH	Cross-national	Research	3	6
Mejdahl et al. 2013 [230]	DK	National	Public Health; Other	2	3
Mellernkjær et al. 2014 [231]	DK	National	Health Services; Public Health	0	1
Messerli et al. 2016 [232]	CH	National	Public Health; Research	0	6
Mikkelsen et al. 2015 [233]	DK	National	Health Services; Other	2	4
Minnerup et al. 2015 [234]	CH	Cross-national	Health Services	0	1
Modvig et al. 2017 [235]	DK	National	Public Health	0	5
Möhring et al. 2019 [236]	CH	Cross-national	Research	1	7
Møller et al. 2008 [237]	DK	National	Public Health	2	6
Mors et al. 2011 [238]	DK	National	Health Services; Public Health	0	9
Mortensen et al. 2011 [239]	DK	National	Public Health; Research	1	3
Mortensen et al. 2013 [240]	DK	National	Health Services; Public Health	0	2
Mueller et al. 2015 [241]	CH	Cross-national	Research	0	7
Mukai et al. 2013 [242]	DK	National	Health Services; Public Health	1	2
Müller et al. 2012 [243]	CH	National	Other	1	4
Munk et al. 2012 [244]	DK	National	Public Health; Other	0	6
Narath et al. 2016 [245]	CH	Cross-national	Health Services; Research	0	7
Neelon et al. 2015[246]	DK	National	Public Health	1	2
Nickenig et al. 2014 [247]	Both	Cross-national	Health Services; Public Health	2	4
Nielsen et al. 2012 [248]	DK	National	Health Services; Public Health	1	3
Nielsen et al. 2015 [249]	DK	National	Health Services; Public Health	2	2
Nielsen et al. 2015b [250]	DK	National	Health Services; Public Health	2	3
Nielsen and Nordestgaard 2016 [251]	DK	National	Health Services; Public Health; Other	1	3
Nilsson et al. 2014 [252]	DK	National	Health Services; Public Health	3	5
Nolan-Kenney et al. 2019 [253]	CH	Cross-national	Research	4	8
Nørskov et al. 2015 [254]	DK	National	Public Health	1	4
Nørskov et al. 2017 [255]	DK	National	Public Health; Research	1	4
Nyholm et al. 2015 [256]	DK	National	Health Services; Public Health	2	3
Olsen et al. 2008 [257]	DK	National	Health Services; Public Health; Other	3	4
Olsen et al. 2013 [258]	DK	National	Public Health	1	5
Orsted et al. 2011[259]	DK	National	Public Health	2	5
Özcan et al. 2016 [260]	DK	National	Health Services	2	10
Pacurariu et al. 2015 [261]	DK	Cross-national	Public Health	5	2
Pagh et al. 2013 [262]	DK	National	Health Services; Other	1	2
Palnum et al. 2012 [263]	DK	National	Health Services; Public Health; Other	2	6
Pasternak et al. 2014 [264]	DK	National	Health Services; Public Health	1	5
Patadia et al. 2018 [265]	DK	Cross-national	Health Services; Research	1	2
Pattaro et al. 2016 [266]	Both	Cross-national	Research	1	7
Paulsen et al. 2013 [267]	DK	National	Public Health	1	6
Pechmann et al. 2019 [268]	CH	Cross-national	Research	1	12
Pedersen et al. 2010 [269]	DK	Cross-national	Health Services	0	2
Pedersen 2011 [270]	DK	National	Health Services; Public Health	2	6
Pedersen et al. 2011 [271]	DK	National	Health Services; Public Health	0	4
Perera et al. 2018 [272]	DK	Cross-national	Public Health	3	1
Perregaard et al. 2015 [273]	DK	National	Public Health	2	3
Petersen et al. 2018 [274]	DK	National	Public Health	3	2
Petersen et al. 2018b [275]	DK	National	Health Services; Public Health; Other	1	3
Piazza et al. 2010 [276]	CH	Cross-national	Health Services	0	1
Piltoft et al. 2017 [277]	DK	National	Public Health; Other	0	4
Pinborg et al. 2015 [278]	DK	National	Public Health	0	4
Pironi et al. 2017 [279]	DK	Cross-national	Health Services	2	5
Plüss-Suard et al. 2013 [280]	CH	National	Health Services	1	3
Pommergaard et al. 2014 [281]	DK	National	Health Services; Public Health	3	3
Pottegard et al. 2014 [282]	DK	National	Public Health	0	9
Pottegard et al. 2015 [283]	DK	National	Public Health	2	6
Poulsen et al. 2012 [284]	DK	National	Health Services	2	4
Poulsen et al. 2016 [285]	DK	National	Health Services; Public Health	1	4
Poulsen et al. 2018 [286]	DK	National	Health Services; Public Health	1	6
Preston et al. 2014 [287]	DK	National	Public Health	0	5
Prins et al. 2018 [288]	DK	Cross-national	Research	0	6
Pukkala et al. 2009 [289]	DK	Cross-national	Public Health	2	6
Radovanovic and Erne 2010 [290]	CH	National	Health Services	3	12
Ramlau-Hansen et al. 2009 [291]	DK	National	Health Services; Public Health	2	3
Rasmussen et al. 2012 [292]	DK	National	Public Health	1	2
Rasmussen and Tønnesen 2016 [293]	DK	National	Public Health; Other	1	7
Rasmussen et al. 2017 [294]	DK	National	Public Health; Other	2	7
Rathe 2015 [295]	DK	National	Health Services; Public Health	0	7
Reyes et al. 2016 [296]	DK	Cross-national	Public Health	0	4
Ringdal et al. 2011 [297]	Both	Cross-national	Research; Other	6	8
Roberto et al. 2016 [298]	DK	Cross-national	Health Services; Public Health; Research	2	8
Rudin et al. 2008 [299]	CH	National	Research	0	5
Rungby et al. 2017 [300]	DK	National	Health Services; Public Health	2	6
Russell et al. 2018 [301]	DK	Cross-national	Research	2	5
Schaefer et al. 2013 [302]	CH	National	Health Services	0	4
Schäfer et al. 2018 [303]	CH	Cross-national	Research; Other	2	6
Schatlo et al. 2012 [304]	CH	National	Health Services; Research	0	4
Schatorjé et al. 2014 [305]	CH	Cross-national	Research	4	6
Schmaal et al. 2017 [306]	CH	Cross-national	Health Services	1	6
Schmidt et al. 2010 [307]	DK	National	Public Health; Other	0	4
Schmidt et al. 2010b [308]	DK	National	Public Health	0	8
Schmidt et al. 2011 [309]	DK	National	Public Health	0	4
Schmidt et al. 2012 [310]	DK	National	Public Health	0	5
Schmidt et al. 2012b [311]	DK	National	Public Health	2	5
Schmidt et al. 2014 [312]	DK	National	Public Health; Other	1	11
Schmidt et al. 2018 [313]	DK	National	Health Services	1	11
Schneeberger et al. 2013 [314]	Both	Cross-national	Health Services	0	3
Schoos et al. 2015 [315]	DK	National	Public Health; Other	0	4
Schroll et al. 2012 [316]	DK	National	Health Services	1	4
Schuemie et al. 2012 [317]	DK	Cross-national	Health Services; Public Health	2	6
Sejbaek et al. 2013 [318]	DK	National	Public Health; Research	0	4
Skyum et al. 2018 [319]	DK	National	Health Services; Other	3	4
Skyum et al. 2019 [320]	DK	Cross-national	Health Services; Research	2	7
Soerensen et al. 2014 [321]	DK	National	Health Services; Public Health	1	7
Sommer et al. 2018 [322]	CH	National	Research	2	6
Sørensen et al. 2009 [323]	DK	National	Public Health	1	1
Sørensen et al. 2013 [324]	DK	National	Health Services; Public Health	1	6
Spoerri et al. 2010 [325]	CH	National	Public Health	1	3
Stahl Madsen et al. 2014 [326]	DK	National	Health Services	0	3
Steenholdt et al. 2014 [327]	DK	National	Public Health; Research	1	6
Stewardson et al. 2016 [328]	CH	Cross-national	Health Services	1	9
Strasser et al. 2016 [329]	CH	National	Research	1	7
Streit et al. 2014 [330]	CH	National	Health Services	2	4
Strnad et al. 2016 [331]	CH	Cross-national	Research	0	6
Stukalin et al. 2018 [332]	DK	Cross-national	Health Services; Public Health	1	3
Sürder et al. 2013 [333]	CH	National	Research	1	4
Suttorp et al. 2018 [334]	CH	Cross-national	Research	0	6
Svendsen et al. 2013 [335]	DK	National	Health Services; Public Health	0	2
Talman et al. 2008 [336]	DK	National	Health Services; Public Health	0	3
Thillemann et al. 2009 [337]	DK	National	Public Health	0	4
Thomsen et al. 2008 [338]	DK	National	Health Services; Public Health	0	5
Thornqvist et al. 2014 [339]	DK	National	Health Services; Public Health	2	6
Thøstesen et al. 2015 [340]	DK	National	Public Health; Research; Other	0	4
Thygesen et al. 2011 [341]	DK	National	Health Services; Public Health	0	5
Tollånes et al. 2016 [342]	DK	Cross-national	Health Services; Public Health; Research	2	7
Trabert et al. 2014 [343]	DK	Cross-national	Research	4	4
Tutolo et al. 2019 [344]	CH	Cross-national	Health Services	1	3
Tvedskov et al. 2011 [345]	DK	National	Health Services; Public Health	2	5
Tvedskov et al. 2015 [346]	DK	National	Health Services; Public Health	1	5
Ulff-Moller et al. 2018 [347]	DK	National	Health Services; Public Health; Research; Other	2	6
Underbjerg et al. 2013 [348]	DK	National	Health Services; Public Health	2	6
Underbjerg et al. 2015 [349]	DK	National	Public Health	0	5
Ungaro et al. 2019 [350]	DK	Cross-national	Public Health	3	6
Usvyat et al. 2013 [351]	Both	Cross-national	Health Services	8	4
Vach et al. 2018 [352]	CH	National	Health Services; Research; Other	2	4
Van Hedel et al. 2018 [353]	CH	Cross-national	Health Services	3	7
Van Stralen et al. 2011 [354]	Both	Cross-national	Research	1	3
Vasan et al. 2016 [355]	DK	Cross-national	Public Health	0	5
Vester-Andersen et al. 2014 [356]	DK	National	Health Services; Public Health	0	6
Vest-Hansen et al. 2014 [357]	DK	National	Public Health	1	5
Viberg et al. 2018 [358]	DK	National	Health Services; Public Health	0	5
Villadsen et al. 2011 [359]	DK	National	Health Services; Public Health	2	3
Walters et al. 2013 [360]	DK	Cross-national	Public Health	5	4
Weber et al. 2013 [361]	CH	National	Health Services	11	5
Weigang et al. 2010 [362]	CH	Cross-national	Health Services	1	3
Wiegand et al. 2014 [363]	CH	Cross-national	Research	1	6
Wildgaard et al. 2011 [364]	DK	National	Health Services; Public Health	1	2
Winterfeld et al. 2013 [365]	Both	Cross-national	Health Services	1	4
Wurtzen et al. 2013 [366]	DK	National	Health Services; Research	0	6
Ylijoki-Sorensen et al. 2014 [367]	DK	Cross-national	Public Health	4	4
Zalfani et al. 2012 [368]	CH	National	Health Services; Public Health	0	4
Zecca et al. 2018 [369]	CH	National	Health Services	2	4
Zellweger et al. 2014 [370]	CH	National	Health Services; Other	2	5
Zellweger et al. 2019 [371]	CH	National	Public Health	1	5
Zwisler et al. 2016 [372]	DK	National	Health Services; Public Health	0	10

^a^ DK: Denmark

^b^ CH: Switzerland

### Overview of barriers

Barriers of an ethical nature were reported 19 times in the included records and they concerned mainly issues related to privacy (n = 9) and respect for autonomy of study participants (n = 6) (Table 3). As to legal barriers, these were reported 17 times and they included issues associated with national data protection regulations (n = 4), differences in national legislations concerning data security and privacy (n = 4) and “Other” (n = 9) (e.g. legal uncertainty concerning health data collection or sharing, market restriction, etc.). Overall, the type of barriers that were more often reported, however, were those of a technical nature. In the records, 416 technical barriers were mentioned and they were classified as data quality issues (e.g. data incompleteness, potential misclassification of data, etc.) (n = 234), lack of data standards (data structure and semantics, e.g. ambiguous terminologies, temporal evolution of data standards, etc.) (n = 151), limited technical capabilities (e.g. no unique identifier, etc.) (n = 21) and “Other” (n = 10) (e.g. time constraints on physicians preventing the use of standard procedures for data collection). Financial barriers were also reported, but only a limited amount of times (n = 9), and they were principally referring to the unavailability or inadequacy of financial support (n = 8). Only 13 political barriers were found and they comprised institutional/constitutional organization issues (e.g. federalist system and different healthcare systems) (n = 6), mistrust between stakeholders (n = 3), data ownership issues (n = 2) and “Other” (n = 2) (e.g. no official guidelines for data sharing). Studies also reported some motivational barriers, including lack of research incentives (n = 17) (including additional workload imposed on physicians/researchers), data re-use prevented by stakeholders as they are deemed unfit for secondary use (n = 2), stakeholders’ competing interests (n = 2) and additional barriers of a diversified content, thus labelled as “Other” (n = 4) (e.g. study participants not showing up for part of the study). Finally, 6 socio-cultural barriers were reported in the included records, half of which were related to a “cultural clash” (n = 3), which we defined as issues resulting from different cultures in data collection, sharing and linkage of the partners involved in the project.

**Table 3:** Distribution of barriers’ sub-clusters in national and cross-national Danish and Swiss projects.

Barriers		Countries involved in projects		
Cluster	Sub-cluster	Denmark N^a^ = 251	Switzerland N = 80	Both countries N = 14
		n^b^ (mean no. of barriers per project)	n (mean no. of barriers per project)	n (mean no. of barriers per project)
**Ethical**	Privacy	6 (0.02)	3 (0.04)	-^c^ (N/A)
	Respect for Autonomy	3 (0.01)	3 (0.04)	- (N/A)
	Other	3 (0.01)	1 (0.01)	- (N/A)
**Legal**	Data Protection Regulations	2 (0.01)	1 (0.01)	1 (0.07)
	Divergence in National Legislations for Data Security and Privacy	2 (0.01)	- (N/A)	2 (0.14)
	Other	5 (0.02)	3 (0.04)	1 (0.07)
**Technical**	Lack of Data Standards	104 (0.41)	33 (0.41)	14 (1.00)
	Data Quality Issues	181 (0.72)	44 (0.55)	9 (0.64)
	Limited Technical Capabilities	11 (0.04)	9 (0.11)	1(0.07)
	Other	8 (0.03)	2 (0.03)	- (N/A)
**Financial**	Lack of Funding	4 (0.02)	3 (0.04)	1 (0.07)
	Other	1 (0.00)	- (N/A)	- (N/A)
**Political**	Mistrust between stakeholders	- (N/A)	3 (0.04)	- (N/A)
	Data Ownership	2 (0.01)	- (N/A)	- (N/A)
	Institutional/constitutional organization issues	2 (0.01)	4 (0.05)	- (N/A)
	Other	- (N/A)	2 (0.03)	- (N/A)
**Motivational**	Lack of research incentives	6 (0.02)	9 (0.11)	2 (0.14)
	Stakeholder restricts access for re-use of data as deemed unfit for secondary use	2 (0.01)	- (N/A)	- (N/A)
	Stakeholder competing interests	1 (0.00)	1 (0.01)	- (N/A)
	Other	1 (0.00)	3 (0.04)	- (N/A)
**Sociocultural**	Cultural clash for data collection/sharing/linkage	1 (0.00)	2 (0.03)	- (N/A)
	Other	1 (0.00)	2 (0.03)	- (N/A)

Table 3 shows the distribution of barriers’ sub-clusters in national and cross-national Danish and Swiss projects. As such, single-country and multi-national countries are not differentiated.

^a^ N is the total number of projects in each country category

^b^ n is the total number of reported barriers per sub-cluster

c–is the absence of reported barriers per sub-cluster

N/A–Not Applicable

### Overview of facilitators

Facilitators of an ethico-legal nature were reported 582 times in total, and they were classified as official/legal approval of study (e.g. Danish Data Protection Agency) (n = 148), ethical approval by a REC/IRB (n = 135), legislation permitting to proceed with health data collection, sharing and linkage without consent or REC/IRB approval (n = 79), obtaining informed consent from participants (n = 69), health data anonymization (n = 58), the presence of legislation requiring mandatory reporting (n = 41), confidentiality measures (n = 29; e.g. data security audits), project done according to international laws and regulations (n = 8), data access rights for patients (n = 4), clear legislation for data collection, sharing or linkage (n = 3) and “Other” (n = 8) (e.g. study data made available by researchers upon request). Facilitators of a technical nature were reported 981 times in total, which were grouped in three categories, namely techniques for data harmonization (n = 798), data linkage (n = 155) and “Other” (n = 28) (e.g. study allowed the creation of optional and mandatory datasets, whereby a minimum of data are classified as mandatory). Facilitators of a financial nature, especially explaining how funding was successfully secured, were mentioned 12 times. These referred, for example, to public-private partnerships, where both partners would gain some benefits from the collaboration, as a solution for funding issues (n = 3). 169 facilitators related to politics were reported. These referred to the structure of the health system as an advantage for harmonized health data collection, sharing and linkage (n = 139), data access control by the players (n = 11), the presence of a data sharing agreement between the stakeholders (n = 9), building and maintaining stakeholders’ trust for collaboration (n = 7) and “other” (n = 4). There were 14 motivational facilitators, which included monetary incentives to incite researchers/stakeholders to abide by standardized procedures for data handling and management (n = 7), improved data collection tool to ease the workload of researchers/stakeholders for data collection/sharing (n = 3), a memorandum of understanding between partners to ensure collaboration till end of study (n = 2) and “other” (n = 2). Lastly, there were 8 socio-cultural facilitators, which included data subjects controlling access to their data (n = 4) and “Other” (n = 4) (e.g. transparent policies for the participants). Country-wise distribution for all six facilitators categories are presented in Table 4.

**Table 4:** Distribution of facilitators’ sub-clusters in national and cross-national Danish and Swiss projects.

Facilitators		Countries involved in projects		
Cluster	Sub-cluster	Denmark N^a^ = 251	Switzerland N = 80	Both countries N = 14
		n^b^ (mean no. of facilitators per project)	n (mean no. of facilitators per project)	n (mean no. of facilitators per project)
**Ethico-Legal** ^c^	Ethical approval by REC/IRB	73 (0.29)	55 (0.69)	7 (0.50)
	Health Data Anonymization	31 (0.12)	22 (0.28)	5 (0.36)
	Obtaining informed Consent	29 (0.12)	34 (0.43)	6 (0.43)
	Patient data access rights	3 (0.01)	1 (0.01)	-^d^ (N/A)
	Confidentiality measures taken	22 (0.09)	6 (0.08)	1 (0.07)
	Clarity of legislation for health data collection/sharing/linkage	2 (0.01)	1 (0.01)	- (N/A)
	Official/legal approval of project	140 (0.56)	7 (0.09)	1 (0.07)
	Project done according to international laws and regulations	6 (0.02)	1 (0.01)	1 (0.07)
	Legislation allows project without consent or REC approval	66 (0.26)	12 (0.15)	1 (0.07)
	Legislation requires mandatory reporting	40 (0.16)	1 (0.01)	- (N/A)
	Other	6 (0.02)	2 (0.03)	- (N/A)
**Technical**	Data harmonization techniques	488 (1.94)	251 (3.14)	59 (4.21)
	Data Linkage techniques	146 (0.58)	6 (0.08)	3 (0.21)
	Other	24 (0.10)	3 (0.04)	1 (0.07)
**Financial**	Securing funding	6 (0.02)	1 (0.01)	1 (0.07)
	Public-Private partnership	1 (0.00)	2 (0.03)	- (N/A)
	Other	1 (0.00)	- (N/A)	- (N/A)
**Political**	Data Sharing Agreement	1 (0.00)	5 (0.06)	3 (0.21)
	Building and maintaining stakeholder trust	1 (0.00)	4 (0.05)	2 (0.14)
	Data access control	9 (0.04)	2 (0.03)	- (N/A)
	Health System Structure	138 (0.55)	1 (0.01)	- (N/A)
	Other	3 (0.01)	- (N/A)	- (N/A)
**Motivational**	Monetary Incentive	5 (0.02)	2 (0.03)	- (N/A)
	Easing workload through improvement of data collection	1 (0.00)	2 (0.03)	- (N/A)
	Memorandum of understanding to ensure collaboration until end of study	- (N/A)	2 (0.03)	- (N/A)
	Other	- (N/A)	1 (0.01)	1 (0.07)
**Sociocultural**	Participant data access control	2 (0.01)	1 (0.01)	1 (0.07)
	Other	4 (0.02)	- (N/A)	- (N/A)

Table 4 shows the distribution of facilitators’ sub-clusters in national and cross-national Danish and Swiss projects. As such, single-country and multi-national countries are not differentiated.

^a^ N is the total number of projects in each country category

^b^ n is the total number of reported facilitators per sub-cluster

^c^ Ethical and legal facilitators were merged as reported solutions had both an ethical and a legal dimension

d–is the absence of reported facilitators per sub-cluster

N/A–Not Applicable

### Barriers and facilitators identified in national Danish and Swiss projects

When considering only national projects (n = 240) involving either Denmark (N = 200) or Switzerland (N = 40) alone, there were 323 identified barriers and 1234 facilitators. Technical barriers and facilitators were most frequently reported. For comparison purposes and compensation for the imbalances in the number of national projects identified in each country, the absolute numbers and the number of barriers and facilitators per 1,000 national projects for each country are illustrated in Table 5.

**Table 5:** Distribution of barriers and facilitators in national Danish and Swiss projects.

Barrier category	Denmark N^a^ = 200	Switzerland N = 40	Facilitator category	Denmark N = 200	Switzerland N = 40
	n^b^ (no. of barriers per 1,000 projects)	n (no. of barriers per 1,000 projects)		n (no. of facilitators per 1,000 projects)	n (no. of facilitators per 1,000 projects)
**Ethical**	6 (30)	6 (150)	**Ethico-legal**	331 (1655)	82 (2050)
**Legal**	6 (30)	4 (100)			
**Technical**	216 (1080)	51 (1275)	**Technical**	523 (2615)	132 (3300)
**Financial**	3 (15)	2 (50)	**Financial**	8 (40)	2 (40)
**Political**	-^c^ (N/A)	8 (200)	**Political**	134 (670)	6 (150)
**Motivational**	7 (35)	8 (200)	**Motivational**	6 (30)	5 (125)
**Sociocultural**	2 (10)	4 (100)	**Sociocultural**	4 (20)	1 (25)
**Total**	240	83	**Total**	1006	228
**Mean**	1.20	2.08	**Mean**	5.03	5.70

^a^ N is the total number of projects in each country category

^b^ n is the total number of identified barriers or facilitators per cluster

^c^–is the absence of identified barriers and facilitators per cluster

N/A–Not Applicable

Interestingly, the only identified category of barriers which was comparatively almost equally reported in Swiss and Danish single-country projects was that of technical barriers. Otherwise, ethical, legal, financial, motivational and socio-cultural barriers were reported 5.0, 3.3, 3.3, 5.7 and 10.0 times more in Swiss projects than in Danish projects respectively. On the contrary, a Swiss project reported on average more facilitators than a Danish one (only financial facilitators were reported equally in both countries). Ethico-legal, technical, motivational and socio-cultural facilitators were reported 1.2, 1.3, 4.2 and 1.3 times more in Swiss projects than in Danish projects respectively. Only facilitators related to politics were reported 4.5 times more in Danish projects than Swiss projects.

### Barriers and facilitators identified in cross-national Danish and Swiss projects

With respect to cross-national projects (n = 105), there were 182 identified barriers and 532 identified facilitators. Technical barriers and facilitators were more frequently reported than those of another nature. For comparison purposes and compensation for the imbalances in the number of cross-national projects involving each country, the number of barriers and facilitators per 1,000 cross-national projects was calculated (excluding cross-national projects involving both countries) and illustrated in Table 6.

**Table 6:** Distribution of barriers and facilitators in cross-national Danish and Swiss projects.

Barrier category	Denmark N^a^ = 51	Switzerland N = 40	Both countries N = 14	Facilitator category	Denmark N = 51	Switzerland N = 40	Both countries N = 14
	n^b^ (Number of barriers per 1,000 projects)	n (Number of barriers per 1,000 projects)			n (Number of facilitators per 1,000 projects)	n (Number of facilitators per 1,000 projects)	
**Ethical**	6 (118)	1 (25)	- ^c^	**Ethico-legal**	87 (1706)	60 (1500)	22
**Legal**	3 (59)	- (N/A)	4				
**Technical**	88 (1725)	37 (925)	24	**Technical**	135 (2647)	128 (3200)	63
**Financial**	2 (39)	1 (25)	1	**Financial**	- (N/A)	1 (25)	1
**Political**	4 (78)	1 (25)	-	**Political**	18 (353)	6 (150)	5
**Motivational**	3 (59)	5 (125)	2	**Motivational**	- (N/A)	2 (50)	1
**Sociocultural**	- (N/A)	- (N/A)	-	**Sociocultural**	2 (39)	- (N/A)	1
**Total**	106	45	31	**Total**	242	197	93
**Mean**	2.08	1.13	2.21	**Mean**	4.75	4.93	6.64

^a^ N is the total number of projects in each country category

^b^ n is the total number of identified barriers or facilitators per cluster

c–is the absence of identified barriers and facilitators per cluster

N/A–Not Applicable

Concerning cross-national projects, we observed a reverse tendency as compared to national projects. Studies involving a collaboration with a Swiss partner have, on average, reported 1.8 times less barriers than those involving a Danish partner. More in detail, projects including Switzerland reported 4.7, 1.9, 1.6, 3.1 times less barriers of an ethical, technical, financial and political nature respectively, than those with a Danish partner. However, cross-national projects involving a Swiss partner, reported 2.1 times more barriers of a motivational nature than those with a Danish partner. Comparatively, cross-national collaboration involving either a Swiss or Danish partner reported almost the same number of facilitators. Ethico-legal and political facilitators were identified 1.1 and 2.4 times more in cross-national projects with a Danish partner as opposed to cross-national projects involving a Swiss one. However, technical facilitators were identified 1.2 times more in cross-national projects with a Swiss partner than in those with a Danish one.

## Discussion

This systematic review provides a comprehensive overview of projects from either Denmark or Switzerland which involved the collection, linking or sharing of data and of the barriers and facilitators related to the usage of health data therein reported. Our study includes a broad range of projects relying on data from different sources and contexts (health services, public health, research and other) and it confirms that studies involving the harmonization, linking or sharing of health data still encounter a high number of obstacles, but also underscores that barriers have prompted the development of numerous solutions. We will here address and discuss the findings related to barriers and facilitators of each cluster that was identified.

### Ethico-legal barriers and facilitators

Although ethico-legal factors are often described as some of the most problematic elements when it comes to linking and sharing health-related data [9, 373, 374], our results show that barriers of this nature are rarely reported. The small amount of ethico-legal barriers identified might either mean that such barriers were rarely present or that they were present but underreported. In our view, the latter option is more probable for at least two reasons. Firstly, as the records included in this review were all published articles, the explicit mentioning of ethico-legal complications might have been avoided to bypass problems related to publication. Secondly, ethico-legal factors are often less tangibile and transparent in comparison–for example–with technical ones [15] and they are thus more likely to be superseded. Moreover, underreporting would confirm that ethico-legal aspects related to processing of health data are still underappreciated, which is a major obstacle to the final success of research projects [2]. This also suggests that there is some resistency by authors to openly disclose and discuss ethico-legal problematics. For the future, a less cautious approach would be much more beneficial, since it would allow new research projects to build on the issues encountered by old ones.

Ethico-legal facilitators were more widely mentioned. Results show that Swiss projects are still predominantly anchored to the “consent or anonymise” approach, according to which the solution to solve ethico-legal problematics concerning health data is to either anonymize information or to require explicit authorization by data subjects [1]. Differently, Danish projects have made vaster use of alternative solutions, such as relying on specific confidentiality tools, and, more importantly, exploiting regulation that allows—upon certain conditions—to share and link health-related data without the need of obtaining consent by data subjects or REC approval. This demonstrates that the development of proper regulations to facilitate the harmonization and linking of health data offers practical solutions that projects developers are then willing to use. In this framework, another important finding concerns the role of the data protection authority. Whereas in Switzerland this public office–although existing–does not play a defined role with respect to research, results show that Danish studies have a more active interaction with the Data Protection Agency, as they need to apply for permission to use health data. The nature of the application to the national Data Protection Agency that Danish projects need to file is not explicitly described in the records reviewed, but it has been presented elsewhere [375, 376] as a less demanding procedure, resembling a simple duty of notification. Thus, many Danish projects dealing exclusively with health data–in accordance with national regulation–do not need to apply for full ethical review from a REC or IRB, an often demanding and lengthy process, but simply have to obtain clearance from the Data Protection Agency. This institutionalized interaction with the public authority responsible to ensure compliance with data processing rules can be an important factor helping project developers, since it incentivizes to proactively tackle privacy concerns. This interaction could thus be considered as a model to inspire changes in the regulatory framework in Switzerland.

### Technical barriers and facilitators

In this systematic review, data quality issues were the most commonly reported barriers, followed by the lack of data standards and limited technical capabilities. Although Denmark has a developed health data infrastructure, numerous identified projects described that data quality problems still affect health services, public health and research datasets [38, 79, 86, 98, 119, 143, 149, 151]. This is confirmed by other studies, such as a review on the Danish National Patient Registry (DNRP) where the authors concluded that data incompleteness and heterogeneous validation methods of data limited the research potential of this registry [377]. Although relevant, data quality issues can be mitigated in a system like the Danish one, since linkage between data from different registries can be easily performed using the personal identification number (CPR) provided to all Danish citizens at birth and to stable residents [270]. Comparatively, Swiss projects and projects involving a Swiss partner also reported slightly more issues related to data quality than to data standards. However, in comparison to their Danish counterparts which reported almost twice more issues related to data quality than data standards, the difference in reporting of data standards and data quality issues was smaller in Swiss projects. This more equivalent reporting could imply that data standard issues are considered as important as data quality issues for the success of Swiss projects. Indeed, the high levels of data-heterogeneity in the Swiss healthcare context might stem from the fragmented nature of the healthcare system, where each of the 26 cantons [federal states] has a high degree of autonomy and where more than 55 health insurers are active [378].

These findings underline how technical issues are interconnected with the context where projects are carried out, and that also external systemic factors–and not simply internal complications of the projects themselves—affect the emerging of these barriers. In Denmark, for example, the presence of nation-wide registries fosters the development of studies relying on secondary use of routinely collected data, where researchers are more likely faced with issues about the quality of data, since the latter was originally collected for a different purpose. On the contrary, in a country like Switzerland—where data are more often prospectively collected—issues about the absence of common standards because of fragmentation are also likely to be evident, on top of data quality issues.

Our findings suggest therefore that even technical issues concerning data are strongly embedded in the surrounding where projects are conceived. This should induce project developers to communicate and learn from each others, since the barriers they will encounter and the solutions they will find are more likely to be dependent also on the context where they act, and not only on the specific features of their research. For example, since Switzerland’s healthcare sector does not use a universal personal identification number because of privacy concerns [379], linkage of data will almost certainly represent a technical challenge, regardless of the features the single project or the data that it aims at using.

### Motivational and financial barriers and facilitators

With respect to motivational and financial factors, our findings are partly in line with the literature. Previous research had underscored that the key motivational and financial aspects concerned the lack of research incentives from resource-limited institutions, the fears of being ‘robbed’ of data before publication or of losing reputation because others might identify errors in the data, the reluctance to facilitate access due to potential inappropriateness of further uses, the need to secure resources for data sharing activities and the necessity to make arrangements between institutions for data management costs [15, 380, 381].

Overall, national and cross-national Swiss projects combined reported more frequently motivational and financial facilitators than their Danish counterparts. This suggests that in a country with a less institutionalised system of data sharing and where studies often have a prospective design, more strategies are elaborated to deal with financial and motivational issues related to data, since–with a lower systemic support–single project developers have to make a greater effort. In contrary, in a context like Denmark—with the high prevalence of studies with retrospective design and the reliance on secondary uses of routinely collected health data–the need for financial and motivational facilitators might be lower. In fact, when health data harmonization is prevalently retrospective, a lower number of actors is involved [382]–since primary data collectors are rarely included–thus reducing the urgency to create motivational or financial incentives for a large number of collaborators.

Another important finding related to financial aspects is that the presence of economic constraints can be the source of additional barriers related to data harmonization, such as data quality issues. For instance, the Swiss project AMIS Plus—concerning a register for acute coronary syndrome—could not envisage systematic site visits to assess data quality or more in-depth questionnaires due to resource limitation [290]. In Denmark, similarly, with the Copenhagen School Health Records Register—a health examination register for schoolchildren containing data on more than 350,000 individuals—financial constraints made it impossible for the authors to computerize the entire health card, thus limiting the understanding of potential confounding variables [47]. This indicates even more that barriers of different natures are interconnected and that new projects need to acknowledge this interconnectedness of the barriers to successfully address them.

### Political barriers and facilitators

Danish national projects did not report any barriers of a political nature, whereas cross-national collaborations mentioned a few, such as data ownership and organizational issues [44, 85, 95]. This suggests that an institutionalization of data processing practices, similar to what occurs in Denmark [383], helps to remove political obstacles. Moreover, the presence of a centralized healthcare system structure also proves helpful, because it reduces the number of actors involved and thus the presence of competing interests. Political issues, however, might re-emerge when projects are cross-national and thus abandon the relatively *safe-haven* created at the national level.

In a context like the Swiss one, on the contrary, political barriers seem to be more relevant for national projects, because these fuel internal conflicts related to the diversity of interests within healthcare and to the difficulty of implementing uniform and centralized policies [132]. In fact, the two most mentioned political facilitators in Switzerland–building trust amongst stakeholders [132, 361] and stakeholders retaining control over data access [132, 290]–are both related to the attempt to coordinate the numerous different parties operating in the health data field and accommodate their competing interests. This might also explain why less political barriers are reported for Swiss cross-national projects. In fact, when projects from a context like the Swiss one go to a supra-national level, the chances of disputes related to in-country political antagonism to emerge is lower.

Our results are thus in line with the literature, where mistrust between stakeholders, absence of comprehensive guidelines for data sharing and lack of legal accountability were identified as major political issues [2, 7, 15]. However, our results further show that the incidence of political barriers seems quite different in single-country studies if compared to cross national ones. This finding is particularly important since it underlines that sometimes the choice of a national or cross-national design might have an impact on the number of political issues encountered.

### Socio-cultural barriers and facilitators

Barriers and facilitators of a socio-cultural nature were rarely mentioned in the included records. Comparatively, the incidence of cultural barriers seems to be higher for Switzerland, where cultural clashes were mentioned more often than for Danish projects. Such difference could be due to the higher degree of fragmentation of the Swiss healthcare system in comparison to the Danish one, which is centralized and state-funded [18]. In fact, one Swiss study [132] reported that the choice for a distributed model in the managing of data was based on prior failures to implement centralized systems of health data and public mistrust towards the concept of centralization. Socio-cultural facilitators were mostly related to the involvement of data-subjects by allowing them to retain control of data access. For instance, the Swiss project reported that data subjects had the possibility to decide which part of their medical records could be considered “stigmatizing”, and thereafter blinded to healthcare professionals, other than their designated and trusted physician [132]. The designated and trusted physician would have access to the full record.

It is naturally impossible to determine whether socio-cultural barriers were actually overlooked or simply not reported. In either case, the limited mentioning of these factors signals an underappreciation of their importance. On the contrary, socio-cultural aspects should be carefully considered by project developers, since the harmonization of health data cannot ignore the cultural peculiarities of the single contexts from where data are pooled [384]. Harmonization, linking and sharing do not happen in a vacuum and opening up the dialogue between data processors and society at large can be an important success factor for the harmonization of health data in the long run.

### Limitations

The limitations of this systematic review include choices that we made regarding the number of databases used for our search, the fact that we did search using English key words, and that only 20 percent of included papers went through double checking for data extraction consistency. We could have thus missed valuable studies that were published only in Danish, French, and German which we could have found if key words had been in those languages. Given the high number of papers included and resources related constraints, we were unable to double check for all information recorded, but in light of low discrepancies found in the portion of records which were double-checked, we are confident in our output. A reporting bias of barriers and facilitators identified in the included papers cannot be excluded as published papers are focused mostly on the effectiveness of their interventions rather than on the implementation phase. It is possible that our results are thus biased towards barriers and facilitators more likely to be reported in the papers (e.g. those of a technical nature). Given the low numbers of certain types of reported barriers and facilitators, it is difficult to compare the situation in the two countries without under- or over-exaggerating their presence or absence in the two countries. However, the main objective of this systematic review was to identify barriers and facilitators to harmonized health data collection, sharing and linkage in Denmark and Switzerland. Causal inference was not part of this review’s primary objectives.

## Conclusion

This systematic review gathered evidence from Switzerland and Denmark to map and describe barriers and facilitators concerning data harmonization, sharing and linkage. Given the focus of this review on Switzerland and Denmark, part of the findings has specific relevance for these two countries. In particular, for Switzerland it has emerged that fragmentation in the health data environment is a key challenge for harmonizing, sharing and linking of data. Since the implementation of more centralized governance systems—which are of great use in Denmark—might not be a viable option for Switzerland because of the political structure of the country, a distributed governance model, which emphasises interoperability of health data, seems to be the preferable way forward. The introduction of Blockchain technology for patient records, which insures security and respects decentralization [385, 386], is reportedly an auspicious technology as its use in the Estonian healthcare system described by Mettler [387] suggests. This review outlined that the existing data infrastructure at the national-level in Denmark incentivizes the completion of retrospective registry-based studies relying on data reuse. Although barriers are still reported, the existence and comprehensiveness of this data infrastructure confirms that past efforts to improve the health data framework have proven successful. For the future, efforts should focus on easing projects involving cross-national collaborations.

However, other findings are meaningful well beyond the borders of the two countries specifically considered. In particular, in this review it has emerged that, although a great number of barriers and facilitators are mentioned by the projects involving health data harmonization, sharing and linking, reporting focusses predominantly on specific aspects–above all technical ones. Whereas technical aspects are certainly important, the reluctancy to mention also issues of other natures is detrimental to the more general effort of the scientific community to favour the harmonization of health data. Referring more openly to the difficulties encountered at the ethico-legal level, for example, might be of help both for new projects to develop appropriate approaches and for policy makers to gather evidence on which regulatory interventions are needed. The under-appreciation of ethico-legal, socio-cultural and other context-specific complexities is a faux-pas, since the trust of both data-subjects and society at large is indispensable for the success a community in improving the health data context, like the experience of Iceland has demonstrated in the past [388]. There, the project to build a national “health sector database” with health information of all citizens imported from their medical records failed also due to the underappreciation of ethico-legal issues (e.g. informed consent and privacy). Specifically, the population complained that inclusion of personal medical records into the database was supposed to happen without consent by individuals or the possibility to opt out. This was felt like a violation of privacy, because of the risk of re-identification and also due to the fact that the database was supposed to be run by a private company [389]. A privacy complaint was brought in front of the national high court, who ruled against the project to build the database. For this reason, the project was definitely aborted [390].

In summary, the success of current and future projects is likely to depend on a better understanding and appreciation of the complexities associated with harmonizing, sharing and linking health data. In the same line, proposed solutions to harmonization issues should not underestimate the contextual particularities of the country, in which such health data processes occur.

## Supporting information

S1 Text PRISMA checklist. (DOCX)
